# Low pretherapy skeletal muscle mass index is associated with an increased risk of febrile neutropenia in patients with esophageal cancer receiving docetaxel + cisplatin + 5-fluorouracil (DCF) therapy

**DOI:** 10.1007/s00520-023-07609-6

**Published:** 2023-02-04

**Authors:** Katsuhiko Nara, Takehito Yamamoto, Yasuyoshi Sato, Koichi Yagi, Koichiro Kawasaki, Tetsuro Toriumi, Tappei Takada, Yasuyuki Seto, Hiroshi Suzuki

**Affiliations:** 1 Department of Pharmacy, The University of Tokyo Hospital, 7-3-1 Hongo, Bunkyo-ku, Tokyo, 113-8655 Japan; 2 The Education Center for Clinical Pharmacy, Graduate School of Pharmaceutical Sciences, The University of Tokyo, Tokyo, Japan; 3 Department of Gastrointestinal Surgery, Graduate School of Medicine, The University of Tokyo, Tokyo, Japan

**Keywords:** Skeletal muscle mass index, Febrile neutropenia, DCF therapy, Bioelectrical impedance analysis

## Abstract

**Purpose:**

Docetaxel + cisplatin + 5-fluorouracil (DCF) therapy, a frequently prescribed regimen for esophageal cancer, is associated with a high risk of febrile neutropenia (FN). This study investigated whether a low skeletal muscle mass index (SMI) is an independent risk factor for FN.

**Methods:**

This retrospective, observational study investigated the SMI of patients with esophageal cancer who received DCF therapy between March 2018 and July 2020. Based on the Asian sarcopenia criteria, patients were divided into two groups: high and low SMI (SMI of < 7.0 and 5.7 kg/m^2^ for males and females, respectively). The incidence of FN was then compared between the two groups.

**Results:**

Thirty-nine patients (20 and 19 in the high- and low-SMI groups, respectively) were included in this study. The incidence of FN was significantly higher in the low-SMI group (63.2% vs. 20.0%, *P* = 0.006). Univariable and multivariable logistic regression analyses revealed that a low SMI was an independent risk factor for FN (odds ratio, 7.178; 95% confidence interval, 1.272–40.507; *P* = 0.026). In addition, the frequency of dose reduction in DCF therapy was significantly higher in the low-SMI group (68.4% vs. 35.0%, *P* = 0.037).

**Conclusion:**

Low SMI is an independent risk factor for FN in patients with esophageal cancer receiving DCF therapy.

**Supplementary Information:**

The online version contains supplementary material available at 10.1007/s00520-023-07609-6.

## Introduction

Esophageal cancer is highly metastatic and has a poor prognosis. Moreover, esophageal cancer is susceptible to lymph node metastasis even in the early stages owing to high-lymph fluid flow through the esophagus [1]. Preoperative chemo(radio)therapy has been adopted worldwide to treat advanced esophageal cancer (stage II/III). In Western countries, chemoradiotherapy (chemotherapy + radiation) is commonly employed as a preoperative therapy [2, 3], whereas chemotherapy alone is widely used in Japan [4]. The cisplatin (CDDP) + 5-fluorouracil (5-FU) therapy (FP therapy) has been recommended and employed as preoperative chemotherapy in Japan. However, owing to its poor 5-year survival rate (54.5%) [5], docetaxel (DOC) + CDDP + 5-FU therapy (DCF therapy) has been introduced as an alternative to FP therapy in recent years. Besides its preferable efficacy, DCF therapy is associated with a high risk of adverse reactions [6, 7]. Among the various adverse reactions, febrile neutropenia (FN) is observed at high frequencies (approximately 20–40%) without prophylactic antibiotics and/or granulocyte-colony stimulating factor with primary prophylaxis [8–13]. Although the current guidelines do not uniformly recommend the prophylactic administration of pegfilgrastim (PEG-G) to patients receiving DCF therapy, several reports have suggested the efficacy of prophylactic PEG-G during DCF therapy [13–15]. FN is associated with a decrease in the relative dose intensity (RDI) and subsequent poor prognosis [16–19]. Therefore, risk factors for FN during DCF therapy have been extensively studied, and age, solitary life, and non-use of PEG-G have been identified as risk factors for FN [15, 20]. However, not all patients with these reported risk factors develop FN, suggesting that additional risk factors need to be explored.

Sarcopenia, defined as “age-related loss of skeletal muscle mass (SMM) with loss of muscle strength and/or reduced physical performance” [21], has been gaining attention as a predictive factor for the efficacy and safety of chemotherapy [22–24]. Patients with esophageal cancer are at a high risk of sarcopenia because malnutrition due to the loss of oral food intake is often observed [25]. Although several studies have investigated the impact of sarcopenia (loss of SMM) on the safety and efficacy of DCF therapy in patients with esophageal cancer [26, 27], the results are controversial. For instance, Kamitani et al. [26] reported that SMM loss is associated with worsening of overall survival (OS) and disease-specific survival (DSS) in patients treated with DCF therapy, whereas Miyata et al. [27] reported that low SMM was not a significant risk factor for developing FN. In recent years, the SMM index (SMI), calculated by dividing appendicular SMM by the square of height, has been adopted as one of the criteria for sarcopenia in the latest version of the Asian Working Group for Sarcopenia 2019 (AWGS 2019) diagnostic criteria [21], and SMI of 7.0 kg/m^2^ (5.7 kg/m^2^ for female) is recommended as the cutoff value. Considering these circumstances, SMI is expected to be widely used in clinical settings. However, to the best of our knowledge, whether SMI is associated with an increased risk of FN has not been elucidated.

This study is aimed at assessing the impact of SMI measured before DCF therapy on FN risk. Although dual-energy X-ray absorptiometry (DEXA) or computed tomography (CT) has been used as the gold standard for measuring SMI in previous studies, we employed bioelectrical impedance analysis (BIA) to measure SMI because of the simplicity of the measurement. SMI measured by BIA is reportedly favorably consistent with that measured using DEXA or CT [28–30]. Furthermore, the latest version of the AWGS 2019 diagnostic criteria [21] adopts BIA as a method for measuring SMI along with DEXA.

## Patients and methods

### Patients

This study included patients with esophageal cancer who received DCF therapy and whose SMI was measured at The University of Tokyo Hospital between March 2018 and July 2020. The exclusion criteria were as follows: (1) patients who were enrolled in other prospective, interventional clinical trials; (2) patients who started chemotherapy > 45 days after the last SMI measurement; (3) patients whose CDDP dosage was lower than the appropriately adjusted dosage depending on the creatinine clearance (Ccr) calculated using the Cockcroft and Gault equation [31] (30 mL/min ≤ Ccr < 40 mL/min, 35 mg/m^2^; 40 mL/min ≤ Ccr < 50 mL/min, 50 mg/m^2^; 50 mL/min ≤ Ccr < 60 mL/min, 60 mg/m^2^; 60 mL/min ≤ Ccr, 70 mg/m^2^); and (4) patients whose chemotherapy was discontinued for reasons other than adverse reactions related to DCF therapy.

### Treatment and supportive care

DCF therapy consisted of intravenous infusion of DOC (70 mg/m^2^) for 1 h, followed by intravenous infusion of CDDP (70 mg/m^2^; dosage was adjusted according to Ccr) for 2 h on day 1, and continuous intravenous infusion of 5-FU (700 mg/m^2^) from days 1 to 5. In this study, one cycle of 21 days or 28 days was adopted for the purpose of neoadjuvant chemotherapy (NAC) or other purposes (e.g., treatment for unresectable/recurrent esophageal cancer, hereafter referred to as “non-NAC”), respectively. Oral levofloxacin (500 mg; dosage adjusted according to Ccr) was administered to all patients from days 5 to 15 as prophylaxis for FN. MgSO_4_ (20 mEq) was administered before CDDP administration, and adequate hydration with normal saline was administered [32], to avoid CDDP-induced renal dysfunction. In our institute, PEG-G is administered on day 7 of DCF therapy because the G-CSF guideline [33] recommends administering it 24 h after anticancer drug administration.

The antiemetic agents used were neurokinin 1 (NK_1_) receptor antagonist ((fos)aprepitant), serotonin (5-hydroxytryptamine)-3 (5-HT_3_) receptor antagonist (palonosetron), and dexamethasone [34]. The patients received oral aprepitant (125 mg) or intravenous fosaprepitant (150 mg) on day 1. Intravenous palonosetron (0.75 mg) and dexamethasone (9.9 mg) were administered on day 1, followed by intravenous dexamethasone (6.6 mg) on days 2–5. The patients also received oral aprepitant (80 mg) on days 2 and 3 when they received oral aprepitant on day 1.

### Data collection and definition

The database comprised the following patient characteristics: age, sex, height, body weight (BW), body mass index (BMI), the purpose of chemotherapy, family structure, distant metastasis, supportive care, reported FN risk factors, laboratory data, adverse reactions, and dose reduction/discontinuation. FN was defined as (1) an axillary temperature of ≥ 37.5 °C and an absolute neutrophil count (ANC) of < 500 cells/µL, or (2) an axillary temperature of ≥ 37.5 °C and an ANC of < 1000 cells/µL with an expected decline to < 500 cells/µL or less within 48 h, according to practical clinical guidelines [35]. In this study, we diagnosed patients with an axillary temperature of ≥ 37.5 °C and an ANC of 500–1000 cells/µL as having FN only after we had confirmed that the attending physician had made an FN diagnosis. Other adverse reactions were evaluated using CTCAE version 5.0.

### Measurement of skeletal muscle mass index

The body composition parameters of the patients (SMM, appendicular SMM, and lean body mass (LBM)) were measured before the initial DCF therapy using BIA (InBody 770 body composition analyzer, Biospace, Tokyo, Japan). SMI was calculated by dividing the appendicular SMM by the square of height. According to the AWGS 2019 criteria [21], the patients were classified into two groups: high-SMI (SMI ≥ 7.0 kg/m^2^ for males or SMI ≥ 5.7 kg/m^2^ for females) group and low-SMI (SMI < 7.0 kg/m^2^ for males or SMI < 5.7 kg/m^2^ for females).

### Statistical analysis

Continuous data are expressed as median (range) unless otherwise mentioned. The Mann–Whitney *U* test was used to compare continuous variables, while the chi-square test was used to analyze categorical data. Fisher’s exact test was used as a replacement for the chi-square test when the expected frequency of one or more cells was < 5. Univariable and multivariable logistic regression analyses were conducted using age (≥ 65 or < 65 years), sex (female or male), purpose of DCF therapy (NAC or non-NAC), Ccr (< 50 mL/min or ≥ 50 mL/min), SMI (low or high), and prophylactic pegfilgrastim (non-user or user) to identify risk factors for developing FN. All tests were two-tailed, and *P* values < 0.05 were considered statistically significant. All statistical analyses were performed using SPSS Statistics for Windows, version 24 (Armonk, NY, IBM Corp.).

## Results

### Background characteristics of patients

**Fig. 1:**
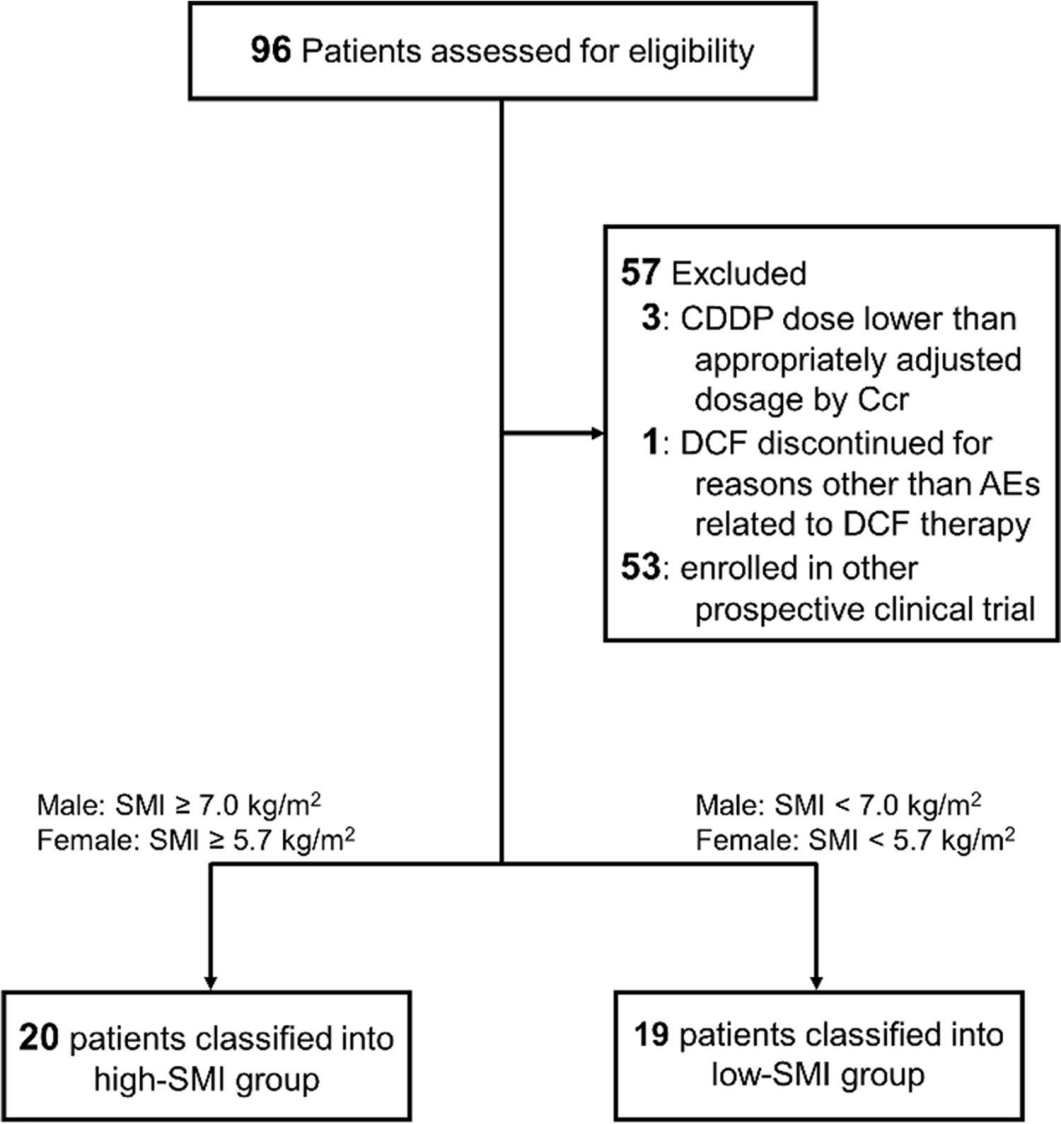
Selection flow of patients in this study

Among the 122 patients who received DCF therapy during the study period, SMI data were available for 96. Fifty-seven patients were excluded, and 39 were included in the analysis. As shown in Fig. 1, 20 and 19 patients were classified into the high- and low-SMI groups, respectively.

Table 1 shows the patients’ backgrounds, laboratory data, body composition parameters, and FN risk factors before DCF therapy. As expected, the body composition parameters (SMM, SMI, and LBM) were significantly lower in the low-SMI group than those in the high-SMI group. In addition, compared to the high-SMI group, the height, BW, BMI, serum albumin (Alb), and hemoglobin (Hb) were significantly lower in the low-SMI group. For other data, no statistically significant differences were observed between the two groups.

**Table 1:** Background characteristics of patients

Characteristics	All patients (*n* = 39)	High-SMI group (*n* = 20)	Low-SMI group (*n* = 19)	*P* value
Age [year], median (range)	68.8 (46.1–84.0)	67.1 (50.8–78.3)	71.2 (46.1–84.0)	0.127*
Sex, male/female (% of male)	28 (71.8)	15 (75.0)	13 (68.4)	0.648^#^
Height [cm], median (range)	164.3 (150.0–180.9)	167.3 (156.0–180.9)	159.2 (150.0–171.0)	0.002*
Body weight [kg], median (range)	55.5 (37.4–86.8)	62.7 (47.9–86.8)	49.2 (37.4–70.7)	< 0.001*
BMI [kg/m^2^], median (range)	20.8 (14.2–29.8)	21.5 (18.9–29.8)	19.5 (14.2–27.0)	0.016*
Purpose of chemotherapy, NAC (%)	22 (56.4)	13 (65.0)	9 (47.4)	0.267^#^
Distant metastasis, *n* (%)	14 (35.9)	8 (40.0)	6 (31.6)	0.584^#^
Cisplatin initial dose reduction, *n* (%)	9 (23.1)	4 (20.0)	5 (26.3)	0.716^##^
Family structure, living alone (%)	12 (30.8)	5 (25.0)	7 (36.8)	0.423^#^
Pegfilgrastim, *n* (%)	21 (53.8)	12 (60.0)	9 (47.4)	0.429^#^
Laboratory data				
Alb [g/dL], median (range)	3.8 (2.4–4.3)	3.9 (2.4–4.3)	3.6 (3.0–4.1)	0.016*
AST [U/L], median (range)	20 (12–51)	22 (14–38)	20 (12–51)	0.901*
ALT [U/L], median (range)	14 (5–56)	16 (8–56)	13 (5–41)	0.184*
ALP [U/L], median (range)	197 (73–1202)	200 (73–350)	191 (110–1202)	0.835*
T-Bil [mg/dL], median (range)	0.6 (0.2–1.3)	0.6 (0.3–0.9)	0.6 (0.2–1.3)	0.607*
Cre [mg/dL], median (range)	0.74 (0.34–1.59)	0.75 (0.53–1.31)	0.71 (0.34–1.59)	0.247*
Ccr [mL/min], median (range)	69.2 (32.6–138.8)	80.9 (43.3–129.3)	67.1 (32.6–138.8)	0.079*
eGFR [mL/min/1.73 m^2^], median (range)	71.5 (34.7–155.5)	70.4 (43.2–97.1)	74.4 (34.7–155.5)	0.351*
CRP [mg/dL], median (range)	0.36 (0.02–13.71)	0.17 (0.03–13.71)	0.68 (0.02–12.22)	0.380*
WBC [/µL], median (range)	6900 (3300–18,200)	7400 (3300–18,200)	6800 (3600–12,000)	0.879*
ANCs [/µL], median (range)	4900 (2100–15,900)	5100 (2100–15,900)	4400 (2500–10,700)	0.687*
Plt [10^4^/µL], median (range)	27.0 (10.9–56.0)	28.5 (10.9–49.8)	26.5 (14.5–56.0)	0.667*
Hb [g/dL], median (range)	12.4 (9.7–16.0)	13.3 (9.7–16.0)	11.5 (9.9–15.0)	0.004*
ALCs [/µL], median (range)	1100 (500–4800)	1150 (600–2100)	1000 (500–4800)	0.647*
Body composition parameter				
Skeletal muscle mass [kg], median (range)	24.1 (16.3–30.6)	27.3 (18.5–30.6)	20.3 (16.3–29.0)	< 0.001*
Skeletal muscle mass index [kg/m^2^], median (range)	6.4 (4.9–8.0)	7.4 (5.7–8.0)	6.0 (4.9–6.8)	< 0.001*
Lean body mass [kg], median (range)	43.8 (30.8–55.4)	49.8 (34.6–55.4)	38.1 (30.8–48.7)	< 0.001*
FN risk factors				
Full dose, *n* (%)	30 (76.9)	16 (80.0)	14 (73.7)	0.716^##^
Age ≥ 65 years, *n* (%)	25 (64.1)	11 (55.0)	14 (73.7)	0.224^#^
Performance status ≥ 2, *n* (%)	0 (0.0)	0 (0.0)	0 (0.0)	-
Previous chemotherapy or radiation therapy, *n* (%)	13 (33.3)	5 (25.0)	8 (42.1)	0.257^#^
Preexisting neutropenia (ANC < 1500/µL), *n* (%)	0 (0.0)	0 (0.0)	0 (0.0)	-
Open wounds or recent surgery, *n* (%)	0 (0.0)	0 (0.0)	0 (0.0)	-
Poor nutritional status (Alb < 3.5 mg/dL), *n* (%)	8 (20.5)	3 (15.0)	5 (26.3)	0.451^##^
Poor renal function (Ccr < 50 mL/min), *n* (%)	5 (12.8)	1 (5.0)	4 (21.1)	0.182^##^
Liver dysfunction (T-Bil > 1.5 mg/dL), *n* (%)	0 (0.0)	0 (0.0)	0 (0.0)	-
Cardiovascular disease, *n* (%)	2 (5.1)	1 (5.0)	1 (5.3)	1.000^##^
Multiple comorbid conditions (≥ 3), *n* (%)	5 (12.8)	3 (15.0)	2 (10.5)	1.000^##^
HIV infection, *n* (%)	0 (0.0)	0 (0.0)	0 (0.0)	-

^#^Chi-square test, ^##^Fisher’s exact test, *Mann–Whitney *U* test

*BMI*, body mass index; *Alb*, serum albumin; *AST*, aspartate transaminase; *ALT*, alanine transaminase; *ALP*, alkaline phosphatase; *T-Bil*, total bilirubin; *Cre*, serum creatinine; *Ccr*, creatinine clearance; *CRP*, C-reactive protein; *WBC*, white blood cell count; *ANC*, absolute neutrophil count; *Plt*, platelet count; *Hb*, hemoglobin; *ALC*, absolute lymphocyte count; *FN*, febrile neutropenia; *HIV*, human immunodeficiency virus

### Adverse reactions, dose reduction/discontinuation

Table 2 shows the incidence of adverse reactions to DCF therapy. In this study, none of the patients developed FN grade 4 severity or greater. Compared with the high-SMI group, FN grade 3 (20.0% vs. 63.2%; *P* = 0.006), neutropenia grade 4 (35.0% vs. 73.7%; *P* = 0.015), and anorexia ≥ grade 3 (0.0% vs. 21.1%; *P* = 0.049) were significantly more frequently observed in the low-SMI group. Anorexia ≥ grade 2, nausea, and diarrhea tended to occur slightly more frequently in the low-SMI group, although there were no statistically significant differences between the two groups. Among the 16 patients in this study who developed FN grade 3, 13 (81.3%) developed FN during the first course, whereas the other three patients developed FN during the second or third course.

**Table 2:** Incidence of adverse reactions during DCF therapy

Adverse reactions	All patients (*n* = 39)	High-SMI group (*n* = 20)	Low-SMI group (*n* = 19)	*P* values
Febrile neutropenia grade 3, *n* (%)	16/39 (41.0)	4/20 (20.0)	12/19 (63.2)	0.010^##^
Prophylactic PEG-G (−), *n* (%)	11/18 (61.1)	3/8 (37.5)	8/10 (80.0)	
Prophylactic PEG-G (+), *n* (%)	5/21 (23.8)	1/12 (8.3)	4/9 (44.4)	
Neutropenia grade 4, *n* (%)	21/39 (53.8)	7/20 (35.0)	14/19 (73.7)	0.015^#^
Prophylactic PEG-G (−), *n* (%)	13/18 (72.2)	4/8 (50.0)	9/10 (90.0)	
Prophylactic PEG-G (+), *n* (%)	8/21 (38.1)	3/12 (25.0)	5/9 (55.6)	
Anorexia				
≥ Grade 2, *n* (%)	27/39 (69.2)	11/20 (55.0)	16/19 (84.2)	0.082^##^
≥ Grade 3, *n* (%)	4/39 (10.3)	0/20 (0.0)	4/19 (21.1)	0.047^##^
Nausea				
≥ Grade 2, *n* (%)	8/39 (20.5)	4/20 (20.0)	4/19 (21.1)	1.000^##^
≥ Grade 3, *n* (%)	2/39 (5.1)	0/20 (0.0)	2/19 (10.5)	0.231^##^
Stomatitis ≥ grade 2, *n* (%)	8/39 (20.5)	5/20 (25.0)	3/19 (15.8)	0.695^##^
Diarrhea ≥ grade 2, *n* (%)	5/39 (12.8)	2/20 (10.0)	3/19 (15.8)	0.661^##^
Creatinine increased, *n* (%)	11/39 (28.2)	6/20 (30.0)	5/19 (26.3)	0.798^#^

^#^Chi-square test, ^##^Fisher’s exact test

As FN, a dose-limiting toxicity of DCF therapy, was more frequently observed in the low-SMI group, we compared the proportion of patients whose dose was reduced or discontinued between the high- and low-SMI groups. As shown in Table 3, the proportion of patients who required dose reduction was significantly higher in the low-SMI group (35.0% vs. 68.4%; *P* = 0.037).

**Table 3:** Incidence and reasons for dose reduction/discontinuation

	High-SMI group (*n* = 20)	Low-SMI group (*n* = 19)	*P* values
Dose reduction, *n* (%)	7 (35.0)	13 (68.4)	0.037^#^
Febrile neutropenia, *n*	4	9	
Neutropenia grade 4, *n*	1	3	
Thrombocytopenia grade 4, *n*	0	1	
Anorexia, *n*	0	4	
Nausea, *n*	1	0	
Diarrhea, *n*	0	1	
Creatinine increased, *n*	1	0	
Discontinuation, *n* (%)	0 (0.0)	2 (10.5)	0.231^##^
Febrile neutropenia, *n*	0	1	
Nausea, *n*	0	1	
Diarrhea, *n*	0	1	
Fatigue, *n*	0	1	
Total, *n* (%)	7 (35.0)	15 (78.9)	0.010^##^

^#^Chi-square test, ^##^Fisher’s exact test

### Univariable and multivariable logistic regression analyses

Because patients in the low-SMI group had a significantly higher FN risk, univariable and multivariable logistic regression analyses were performed to assess whether a low SMI was an independent risk factor for FN. As shown in Table 4, univariable and multivariable logistic regression analyses using six factors (age, sex, purpose of chemotherapy, Ccr, SMI, and prophylactic PEG-G use) as covariates revealed that only low SMI was an independent risk factor for FN (odds ratio, 7.178; 95% confidence interval, 1.272–40.507; *P* = 0.026).

**Table 4:** Univariable and multivariable logistic regression analyses of risk factors for FN

Covariates	Univariable analysis		Multivariable analysis	
	Odds ratio (95% CI)	*P* values	Odds ratio (95% CI)	*P* values
Age (≥ 65 years vs. < 65 years)	7.636 (1.406–41.488)	0.019	4.804 (0.640–36.049)	0.127
Sex (female vs. male)	0.762 (0.181–3.211)	0.711	0.776 (0.111–5.425)	0.798
Purpose of chemotherapy (non-NAC vs. NAC)	1.011 (0.279–3.661)	0.987	0.969 (0.175–5.384)	0.972
Ccr (< 50 mL/min vs. ≥ 50 mL/min)	2.423 (0.356–16.499)	0.366	0.597 (0.044–8.041)	0.697
SMI (low SMI vs. high SMI)	6.857 (1.627–28.899)	0.009	7.178 (1.272–40.507)	0.026
Pegfilgrastim (non-use vs. use)	5.029 (1.264–20.002)	0.022	4.189 (0.753–23.322)	0.102

*CI*, confidence interval; *Ccr*, creatinine clearance; *FN*, febrile neutropenia; *NAC*, neoadjuvant chemotherapy; *SMI*, skeletal muscle mass index

## Discussion

The results of this study revealed that low SMI is an independent risk factor for FN in patients with esophageal cancer receiving DCF therapy. In addition, it was also revealed that patients with low SMI are at a higher risk of dose reduction due to adverse reactions related to DCF therapy. To the best of our knowledge, this is the first study to identify low SMI as an independent risk factor for FN in patients with esophageal cancer receiving DCF therapy. We believe that SMI could be useful in predicting the individual risk of FN during DCF therapy in a clinical setting.

As shown in Table 2, 41.0% of the patients (16/39) developed FN, even though 53.8% received PEG-G as primary prophylaxis for FN. In addition, a statistically significant reduction in the incidence of FN was observed in patients receiving prophylactic PEG-G administration (61.1% (11/18) vs. 23.8% (5/21); relative risk reduction, 61%; *P* = 0.026, Table 2). Although a preventive effect of PEG-G was observed in our study, the magnitude of risk reduction was smaller than that in a previous study by Ohkura et al. [15] (3.0% vs. 32.2%; relative risk reduction, 91%). However, when stratified by the SMI of patients, the H-SMI group showed a similar magnitude of decrease in FN incidence (from 37.5 to 8.3%, Table 2) to that observed in the previous study. In contrast, the L-SMI group showed a more moderate decrease in FN incidence after prophylactic PEG-G administration (from 80 to 44%, Table 2). The distribution of SMI in the previous study is unknown, but it is possible that the proportion of patients with L-SMI status in our study was higher than that in the previous study by Ohkura et al. [15], and that this may have led to underestimation of the efficacy of prophylactic PEG-G. Furthermore, patients who received prior chemotherapy or who did not receive a full dose of DCF therapy because of organ dysfunction were excluded in the previous study by Ohkura et al., whereas our study included these patients, which may have increased the incidence of FN and decreased the prophylactic effect of PEG-G.

As shown in Table 2, the incidence of FN was significantly higher in the low-SMI group than that in the high-SMI group (63.2% vs. 20.0%, *P* = 0.006), and multivariate logistic regression analysis identified low SMI as an independent risk factor for FN development (odds ratio 7.178; *P* = 0.026) (Table 4). To the best of our knowledge, this study is the first to identify a low SMI as an independent risk factor for FN. Although the underlying mechanisms of this observed relationship between low SMI and increased risk of FN remain to be elucidated, there are several possible explanations. One is that a low SMI reflects low Alb, which is already known as a risk factor for FN [36, 37]. Second, low SMI is associated with alterations in the pharmacokinetics (PK) of chemotherapeutic agents. From this point of view, several previous reports have indicated that the body composition parameters of patients alter the PK of chemotherapeutic agents. For instance, Gusella et al. [38] reported that the clearance of 5-FU strongly correlates with fat-free mass (FFM) rather than BW and body surface area, and patients with lower FFM exhibited lower 5-FU clearance. In addition, several previous studies have revealed that the LBM-normalized dosage of CDDP and 5-FU was higher in patients with hematological toxicity than in those without hematological toxicity, suggesting that LBM is well correlated with 5-FU clearance [39–41]. Because it has been reported that FFM is roughly equal to LBM [42], and a strong correlation was observed between LBM and SMI in our study (*r*_*s*_ = 0.872, *P* < 0.001, Supplementary Fig. S1 (Online Resource 1)), it is possible that the clearance of 5-FU decreased and its blood concentrations increased in the low-SMI group, and that the increased drug exposure was associated with an increased risk of adverse reactions. Future PK studies are required to fully elucidate the relationship between clearance of chemotherapeutic agents and SMI.

Because low SMI was identified as a risk factor for FN, we compared the proportion of patients who required dose reduction/discontinuation, as FN is a major dose-limiting toxicity during DCF therapy. As shown in Table 3, the proportion of patients with dose reduction was significantly higher in the low-SMI group than that in the high-SMI group (68.4% vs. 35.0%, *P* = 0.037). Considering that the RDI of DCF therapy has been reported to affect long-term prognosis in previous literature [17–19], the higher proportion of patients whose dose was reduced in the low-SMI group may have led to a worse long-term prognosis. Although the relationship between low SMI and long-term prognosis needs to be evaluated in future studies, normalizing SMI by improving nutritional status before DCF therapy may improve safety and long-term prognosis.

Although our study provided clinically important information, it had several limitations that should be considered. First, this was a single-center, retrospective, observational study with a relatively small number of patients; thus, it was impossible to completely exclude unintended biases that may have affected the results. Therefore, the results of this study need to be verified in future studies with larger sample sizes. Second, body composition parameters were measured using the BIA method only, and DEXA or CT, the gold standards for measuring body composition parameters, was not used in this study. Although data obtained by BIA correlate well with those obtained by DEXA or CT [28–30], it is uncertain whether our results can be generalized to SMI data measured by DEXA or CT. Third, this study did not investigate the long-term prognosis, such as OS and DSS. Further studies are needed to clarify the relationship between SMI and long-term prognosis. Fourth, the dose of 5-FU used in this study (700 mg/m^2^) was different from that used in the JCOG 1109 trial (750 mg/m^2^). Therefore, the generalizability of the results of this study to patients who receive 5-FU at a dose of 750 mg/m^2^ should be confirmed in the future studies.

In conclusion, we identified low SMI as an independent risk factor for FN in patients with esophageal cancer receiving DCF therapy. We also revealed that a low SMI was associated with a higher risk of dosage reduction during DCF therapy. Considering the simplicity of measuring SMI using the BIA method, SMI would be a useful predictive factor for FN, and SMI measurement before DCF therapy would increase the safety and efficacy of chemotherapy in patients with esophageal cancer scheduled to receive DCF therapy.

## Supplementary Information

Below is the link to the electronic supplementary material.

Supplementary file1 (PPTX 44 KB)

## Data Availability

The data supporting the findings of this study are available upon request from the corresponding author. The data were not publicly available because of privacy or ethical restrictions.
