# Supplementary material for: Low pretherapy skeletal muscle mass index is associated with an increased risk of febrile neutropenia in patients with esophageal cancer receiving docetaxel + cisplatin + 5-fluorouracil (DCF) therapy
Source: Support Care Cancer. 2023 Feb 4;31(2):150. doi: 10.1007/s00520-023-07609-6 (PMC9898323; doi:10.1007/s00520-023-07609-6)
Supplement: Supplementary file 1 — Supplementary file1 (PPTX 44 KB) [file 520_2023_7609_MOESM1_ESM.pptx]

## Slide 1
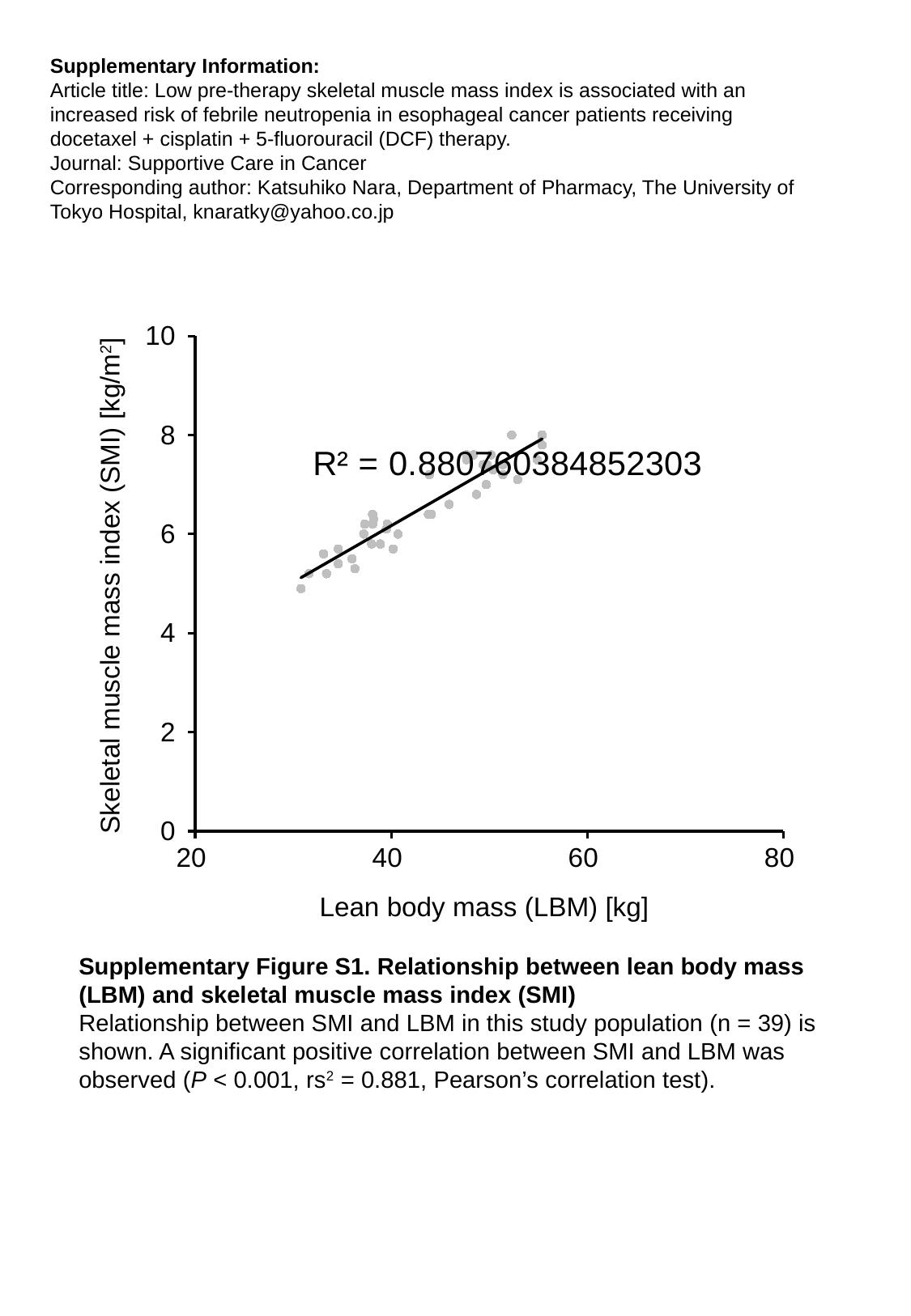

Supplementary Information:
Article title: Low pre-therapy skeletal muscle mass index is associated with an increased risk of febrile neutropenia in esophageal cancer patients receiving docetaxel + cisplatin + 5-fluorouracil (DCF) therapy.
Journal: Supportive Care in Cancer
Corresponding author: Katsuhiko Nara, Department of Pharmacy, The University of Tokyo Hospital, knaratky@yahoo.co.jp
### Chart
| Category | |
|---|---|Skeletal muscle mass index (SMI) [kg/m2]
Lean body mass (LBM) [kg]
Supplementary Figure S1. Relationship between lean body mass (LBM) and skeletal muscle mass index (SMI)
Relationship between SMI and LBM in this study population (n = 39) is shown. A significant positive correlation between SMI and LBM was observed (P < 0.001, rs2 = 0.881, Pearson’s correlation test).
